# Human umbilical cord mesenchymal stem cell derived exosomes (HUCMSC-exos) recovery soluble fms-like tyrosine kinase-1 (sFlt-1)-induced endothelial dysfunction in preeclampsia

**DOI:** 10.1186/s40001-023-01182-8

**Published:** 2023-08-09

**Authors:** Xinwen Chang, Qizhi He, Mengtian Wei, Linyan Jia, Yingying Wei, Yiding Bian, Tao Duan, Kai Wang

**Affiliations:** 1 Center of Reproductive Medicine, Shanghai First Maternity and Infant Hospital, School of Medicine, Tongji University, Shanghai, 201204 China; 2 Department of Pathology, Shanghai First Maternity and Infant Hospital, School of Medicine, Tongji University, Shanghai, 201204 China; 3 Clinical and Translational Research Center, Shanghai Key Laboratory of Maternal Fetal Medicine, Shanghai First Maternity and Infant Hospital, School of Medicine, Tongji University, Shanghai, 201204 China; 4 Department of Obstetrics, Shanghai First Maternity and Infant Hospital, School of Medicine, Tongji University, Shanghai, 201204 China

**Keywords:** Preeclampsia, Mesenchymal stem cell, Exosomes, Angiogenesis, Soluble fms-like tyrosine kinase-1/sFlt-1

## Abstract

**Background:**

Preeclampsia is a unique multisystem disorder that affects 5–8% of pregnancies. A high level of soluble fms-like tyrosine kinase-1 (sFlt-1) is a hallmark of preeclampsia that causes endothelial dysfunction. Exosomes derived from mesenchymal stem cells (MSCs) have been indicated to improve endothelial performances by transporting signals to target cells. We hypothesized that exosomes derived from MSCs have potential effects against preeclampsia.

**Methods:**

We collected human umbilical cord MSC-derived exosomes (HUCMSC-exos) by ultracentrifugation. The size and morphology of the exosomes were examined using a transmission electron microscope and nanoparticle tracking analysis. Pregnant mice were injected with murine sFlt-1 adenovirus to build the preeclampsia-like mouse model and then treated with HUCMSC-exos. Human umbilical vein endothelial cells (HUVECs) were infected with lentiviruses expressing tet-on-sFlt-1 to obtain cells overexpressing sFlt-1. Cell proliferation and migration assays were used to measure the endothelial functions. The exosomes enriched proteins underlying mechanisms were explored by proteomic analysis.

**Results:**

In the current study, we successfully collected the cup-shaped HUCMSC-exos with diameters of 30–150 nm. In the sFlt-1-induced preeclampsia mouse model, HUCMSC-exos exhibited beneficial effects on adverse birth events by decreasing blood pressure and improving fetal birth weight. In addition, preeclamptic dams that were injected with HUCMSC-exos had rebuilt dense placental vascular networks. Furthermore, we observed that HUCMSC-exos partially rescued sFlt-1-induced HUVECs dysfunction in vitro. Proteomics analysis of HUCMSC-exos displayed functional enrichment in biological processes related to vesicle-mediated transport, cell communication, cell migration, and angiogenesis.

**Conclusion:**

We propose that exosomes derived from HUCMSCs contain abundant Versican and play beneficial roles in the birth outcomes of sFlt-1-induced preeclamptic mice by promoting angiogenesis.

**Graphical Abstract:**

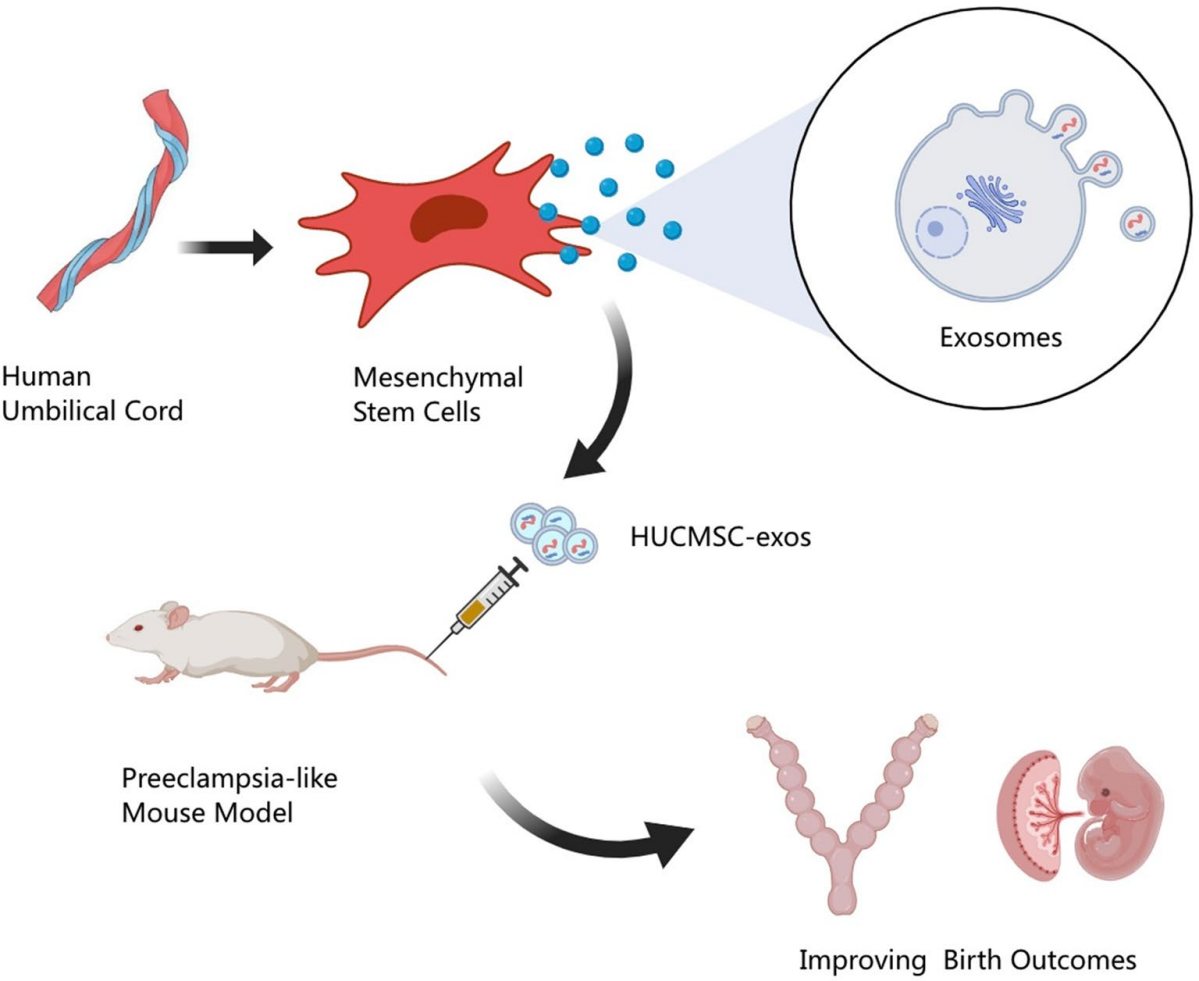

## Introduction

Preeclampsia (PE) is characterized by new-onset hypertension at 20 or more weeks of gestation with or without proteinuria [1]. This gestation-specific syndrome is among the primary causes of maternal, fetal, and neonatal mortality worldwide, and it is also associated with intrauterine growth retardation (IUGR) and preterm birth [2]. An imbalance between proangiogenic and antiangiogenic growth factors, such as soluble fms-like tyrosine kinase-1 (sFlt-1), primarily causes PE, leading to multisystem endothelial dysfunction [3]. Circulating sFlt-1 level is increased in PE and can begin to rise at 21 to 24 weeks of gestation before the onset of clinical symptoms [4]. Women who are diagnosed with PE have decreased quality of life and elevated risk of postpartum depression.

Mesenchymal stem cells (MSCs) are adult cells that possess multipotent properties to differentiate into multiple cell types. Among various types of stem cells, human umbilical cord MSCs (HUCMSCs) have gained prominence as a potential allogeneic cell-based therapy because of their ethical accessibility and rapid renewal properties [5]. In addition, HUCMSCs are considered nonimmunogenic because their low expression of major histocompatibility complex-II (MHC-II) and have, therefore, emerged as one of the most promising treatment options for vascular diseases [6]. HUCMSCs have been under investigation in a variety of clinical therapeutic trials including cardiovascular deficits, recovering ovarian function, and immune system diseases [7]. Our previous work demonstrated that HUCMSCs provided a new avenue for treating placenta-related diseases during pregnancy like PE [8], however, the underlying molecular mechanism is yet to be fully understood.

A large body of evidence suggests that HUCMSCs secrete extracellular vesicles (EVs) containing several functional molecules. Cells release EVs of various sizes, including nanosized extracellular vesicles called exosomes (30–150 nm) that are of endocytic origin [9]. In contrast, microvesicles are larger EVs (150–1000 nm) generated by shedding from the plasma membrane, and there are no specific markers that distinguish them from exosomes [10]. Exosomes are cup-shaped phospholipid nanocarriers, which contain many constituents including DNA, RNA, lipids, metabolites, cytosolic and cell-surface proteins, functioning as transmitting amounts of bioactive molecules for intercellular communication [11]. Exosomes have been well studied in angiogenesis, and our previous research indicated that exosomes from PE patients induced endothelial dysfunction by transferring high levels of sFlt-1 and soluble Endoglin to human umbilical vein endothelial cells (HUVECs) [12].

A concerted effort has been made to reduce the burden of PE, while therapies with the potential to prevent PE progression or reverse disease-induced organ damage are lacking [13]. Currently, delivery is the only definitive treatment, and low-dose aspirin and calcium are recommended for high-risk populations. Pravastatin, metformin, and exercise are currently being investigated [14]. Thus, the pursuit of a treatment option to replace expectant management and instant delivery needs to be investigated. Recently, HUCMSC-derived exosomes (HUCMSC-exos) showed high translational value in anti-aging intervention by enhancing the regenerative capacities in bone formation, wound healing, and angiogenesis [15]. In addition, HUCMSC-exos have been recognized as new candidates for the treatment of vascular diseases because they are selectively enriched in proangiogenic molecules [16]. However, the roles and underlying mechanisms of HUCMSC-exos in the treatment of reproductive complications such as PE are only beginning to be understood and appreciated.

## Materials and methods

### Isolation of HUCMSCs

Human umbilical cord tissues were collected from full-term, healthy cesarean births and were donated by consenting mothers at the Department of Obstetrics of Shanghai First Maternity and Infant Hospital from 2018 to 2019. The demographic and clinical characteristics of the participants in this study were reported in our previous study [17]. The HUCMSCs from human umbilical cord Wharton’s jelly were isolated by the tissue adherence method [18]. Umbilical arteries and veins were removed, and the remaining Wharton’s jelly was transferred to sterile, ice-cold Hank's balanced salt solution (HBSS, Gibco, Thermo Fisher Scientific, Inc., Waltham, MA, USA) containing antibiotics (streptomycin/penicillin, Gibco). The Wharton’s jelly tissues were washed multiple times with HBSS to remove excess blood and were subsequently cut into small pieces. The pieces were transferred to 10 cm^2^ dishes with 5 mL of minimum Eagle’s medium alpha (Gibco) containing 10% fetal bovine serum (FBS, Gibco) and 1% streptomycin/penicillin and were incubated at 37 °C in 5% CO_2_. The explants were left undisturbed for 7–10 days to allow cell migration, and the medium was changed every three days. After 2 weeks, the HUCMSCs (passage 0) were grown to 60–70% confluence and passaged by trypsinization. Sample collection was approved by the Research and Ethics Committee of Shanghai First Maternity and Infant Hospital (No. KS2024).

### Identification of HUCMSCs by flow cytometry and osteogenic differentiation

MSC surface marker detection by a Fluorescence-activated cell sorting (FACS) Calibur Flow Cytometer (BD Biosciences, San Jose, CA, USA) was previously shown [17]. Briefly, 1 × 10^6^ passage 3 cells were trypsinized and suspended in 100 µL of phosphate buffer saline (PBS). Then, the cells were incubated for 30 min with the following monoclonal antibodies against HUCMSCs surface markers: CD45-PE, CD73-APC, CD90-FITC and CD105-PE-Cy7 (BD Biosciences, San Jose, CA, USA).

For osteogenic differentiation, passage 3 cells were cultured in differentiation medium (Gibco) for 3 weeks in six-well plates. After the cells were fixed with 4% paraformaldehyde (PFA) and stained with alizarin red S (Sigma–Aldrich, Merck, Darmstadt, Germany), the cells were observed by microscopy (Nikon Eclipse Ti, Tokyo, Japan).

### Isolation and characteristics of HUCMSC-exos

HUCMSCs at passages 3–6 were cultured in FBS-free medium with 2% bovine serum albumin (BSA, Sigma–Aldrich) for 48 h, and then 500 mL of the supernatant was centrifuged at 3000 g for 10 min to remove cell debris and passed through a 0.22-µm filter. The cleared supernatant was ultracentrifuged at 110,000 g for 70 min and washed in PBS using the same ultracentrifugation conditions to isolate exosomes. Exosome pellets were suspended in 5 mL of PBS and stored at − 80 °C.

The size and morphology of the exosomes were examined using a transmission electron microscope (TEM) at the Laboratory of Electron Microscopy (Chinese Academy of Sciences, Shanghai, China). The size distribution of HUCMSC-exos was determined using nanoparticle tracking analysis (NTA, ZetaView, Particle Metrix, Meerbusch, Germany). These procedures were performed as previously described [12].

### Mice

All mouse experiments were approved by the Department of Laboratory Animal Science, Tongji University (No. TJLAC-020-061). Adult male (*n* = 40) and female (*n* = 80) CD-1 (ICR) mice were purchased from Jackson Laboratories. Animals were housed in a temperature- and humidity-regulated environment with a 12-h light cycle. For a consistent and accurate assessment of the gestational age of mouse embryos, male and female (1:2) mice were pair-housed for one night. Embryonic day 0.5 (E0.5) was designed as the first morning at which a vaginal plug was noted. A well-characterized sFlt-1 overexpression model was established by injecting 6 × 10^8^ pfu of the murine sFlt-1-expressing adenovirus into pregnant ICR mice via the tail vein on E8.5 [19].

Pregnant mice (*n* = 32) were randomly divided into four groups (*n* = 8 per group) and injected with either Cytomegalovirus (CMV)-null adenovirus or murine sFlt-1 adenovirus on E8.5 and HUCMSC-exos (EXO, 100 µg/dam) or sterile saline (NS, 100 µL) on E6.5, E9.5, E12.5 and E15.5 via the tail vein. The groups were designated as follows: (*i*) control (CTL): pregnant mice administered CMV-null adenovirus and NS; (*ii*) EXO: pregnant mice administered CMV-null adenovirus and HUCMSC-exos; (*iii*) sFlt-1: pregnant mice administered sFlt-1 adenovirus and NS; and (*iv*) sFlt-1 + EXO: pregnant mice administered sFlt-1 adenovirus and HUCMSC-exos. In total, there were 4 treatment groups with respect to sFlt-1 overexpression and HUCMSC-exos administration. Blood pressure was noninvasively measured on E18.5 by determining the tail blood volume with a volume pressure recording sensor (CODA System, Kent Scientific, Torrington, CT, USA) that was averaged over a 10-min period. Embryos were harvested on E18.5, and serum, tissues, and urine samples were obtained before euthanasia as described elsewhere [20].

### Tissue preparation for histological analysis

Placental and kidney tissues were fixed with 4% PFA for 48 h and processed by conventional procedures. Sections 3–5 μm in thickness were cut from the paraffin-embedded tissues, mounted on poly-L-lysine-coated slides, deparaffinized in xylene, dehydrated in alcohol and then stained with hematoxylin and eosin (H&E) or periodic acid-methenamine silver (PAS) stain.

### Immunohistochemical (IHC) analysis of mouse placental tissues

For IHC analysis, paraffin sections were deparaffinized and incubated with citrate buffer for antigen retrieval. The slides were then incubated with rabbit anti-CD31 polyclonal antibody (1:100, Abcam, Cambridge, MA, USA) overnight at 4 °C and developed using the ImmPRESS horseradish peroxidase (HRP) anti-rabbit IgG polymer detection kit (Weiao, Shanghai, China).

### Morphological analysis

Using CD31 as an endothelial marker, the densities, diameters, and areas of fetal blood vessels were analyzed as previously described [21]. Four images per tissue section were taken using a Nikon inverted microscope with a 40 × objective (vascular density) or 100 × objective (vascular diameter). For each image, the number of capillaries was counted, and the lumen area was measured using ImageJ imaging analysis software (NIH, Bethesda, MD). Five capillaries per image were randomly selected for diameter measurements by CaseViewer software (3DHISTECH, Ltd., Hungary).

### ELISAs

ELISAs for mouse sFlt-1 and sEndoglin were performed according to the manufacturer’s instructions (R&D Systems). Urine albumin/creatinine ratio was measured using Urinary Albumin and Creatinine Assay kits (Abnova, Taipei City, Taiwan, China). Briefly, the various samples were diluted 1:2 in dilutions and were incubated in a 96-well plate pre-coated with capture antibodies. The wells were washed and incubated with a secondary antibody conjugated to horseradish peroxidase. Then substrate solution was added, and optical density was determined at 450 nm. The concentrations were calculated using a standard curve of the respective recombinant proteins.

### Western blot

Total protein concentration was measured using the bicinchoninic acid assay (Pierce^®^, Thermo Fisher Scientific, Bonn, Germany). Western blot for expressions in exosomes was performed using CD63 (1:1000, System Bioscience, SBI, USA), CD81 (1:1000, SBI), CD9 (1:1000, SBI), sFlt-1 (1:1000, Abcam), Flag (1:2000, Sigma), eNOS (1:1000, Cell Signaling Technology, CST), Versican (1:1000, Abcam), α-Tublin (1:2000, Abmart, Shanghai, China), Flotillin-1 (1:1000, CST) and GAPDH (1:5000, Abmart) antibodies by previously described methodology.

### Cell culture

The HUVECs were isolated from 3 individual donors by a standard collagenase enzyme digestion method and cultured steadily in Endothelial Cell Medium (ECM, ScienCell, San Diego, CA) containing 5% FBS, 1% P/S and 1% endothelial cell growth supplement (ECGS).

### Tet-One induced sFlt-1 expression in HUVECs

The human sFlt-1 (ID: NM_001159920.1) cDNA was cloned into Lenti-X Tet-One System expression vector (Clontech, Moutain View, CA) as described previously. The recombinant lentivirus tet-sFlt-1 and the negative control lentivirus (NC-lentivirus; Hanyin Co. Shanghai, China) were prepared and titered to 10^9^ TU/ml (transfection unit). HUVECs were infected with lentiviruses expressing Tet-on-sFlt-1 to obtain cells overexpressing sFlt-1 only at the time when tetracycline existing. HUVECs expressed the sFlt-1 in the cellular background with doxycycline (Dox) and the efficiency of overexpression was examined by western blot analysis.

### Exosome labelling

Exosomes were labeled with the fluorescent dye 1,10-dioctadecyl-3,3,30,30-tetramethylindocarbocyanine perchlorate (Dil, red, Sigma) by addition to PBS and incubated for 15 min according to the manufacturer’s protocol [22]. The labeled exosome suspensions were filtered using a 100-kDa MWCO hollow fiber membrane (Thermo Fisher Scientific) to remove the excess dye. HUVECs were seeded in 6-well plates and incubated with the Dil-labeled exosomes (100 μg/mL) for 24 h. Then the cells were fixed with 4% paraformaldehyde for 15 min and stained with phalloidin-FITC (Sigma). Nuclei were stained with 4',6-diamidino-2-phenylindole (DAPI, Sigma). The cellular uptake of exosomes was tested by a confocal microscopy (TCS SP8; Leica, Wetzlar, Germany).

### Cell proliferation and migration assays

Cell proliferation and migration ability was performed using modified systems as described previously [23]. In brief, 3 × 10^3^ cells/well were plated in 96-well plates, later Dox or the vehicle were added to induce overexpression of sFlt-1. After 24 h, HUCMSC-exos with different doses were added. Then cell numbers were assessed using the cell counting kit-8 (CCK-8) at 450 nm. For migration, HUVECs were plated in 6-well plates and treated with Dox or the vehicle to induce overexpression of sFlt-1. Then 3 × 10^4^ cells were seeded into the upper chambers and exosomes (100 µg/mL) or NS were added into the lower chambers. Following incubation for 16 h, fluorescent stain (calcein-AM) was added to each lower chamber. The migrated cells were counted by fluorescence analysis (Nikon, Tokyo, Japan).

### Proteomic analysis

The HUCMSCs-exo and HUCMSCs samples were processed for tandem mass tag (TMT)-based quantitative proteomic analysis by Lu-Ming Biotech Co., Ltd. (Shanghai, China). High-performance liquid chromatography tandem mass spectrometry (HPLC–MS/MS) was used to compare the proteomic content of HUCMSCs-exo and cells. Differentially expressed proteins were identified with a cutoff of absolute fold change ≥ 2 and *P*-value < 0.05.

### Statistics

All data were expressed as the mean ± the standard error of the mean (S.E.M) and analyzed using the SPSS 23.0 statistical analysis software (SPSS Inc., Chicago, IL, USA). Statistical significance was determined by performing paired Student’s t-test, one-way analysis of variance (ANOVA) and Dunett’s post-hoc test. A *p*-value < 0.05 was considered statistically significant.

## Results

### Isolation and characterization of HUCMSC-exos

**Fig. 1:**
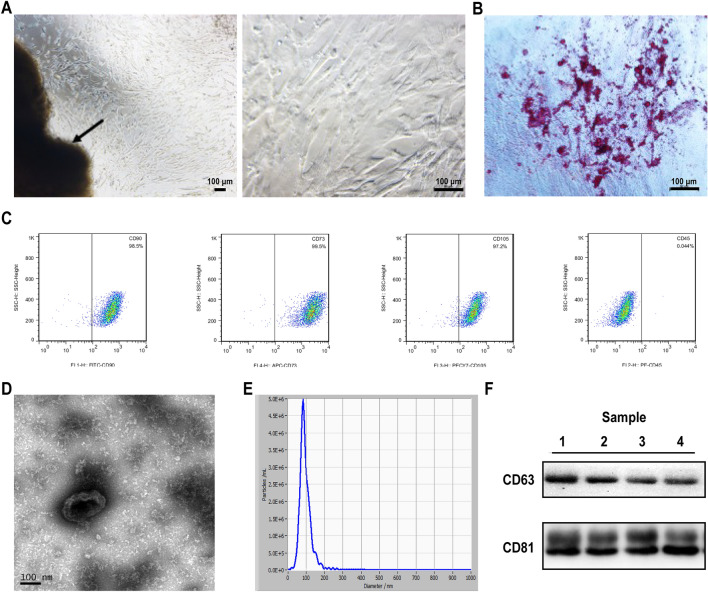
Identification of HUCMSCs and their derived exosomes. **a** Cell morphology under an inverted microscope, scale bar, arrows indicated the tissue section. **b** Osteogenic differentiation of hUCMSCs stained positive with alizarin red. **c** Identification of HUCMSCs by flow cytometry for CD90, CD73, CD105 (positive markers), and CD45 (negative marker). **d**–**f** Exosomes collected from HUCMSCs supernatant by ultracentrifuge. Electron micrograph of exosomes. (**d**). Representative vesicle size (nm) distribution by NTA (**e**). Western blot analysis for the CD63 and CD81 exosomes enriched marker of HUCMSCs-exo from 4 individual pregnant women (**f**)

We isolated HUCMSCs from human umbilical cord Wharton’s jelly using the tissue adherence method. Observation under an inverted microscope showed that the HUCMSCs (passage 0) had long fusiform shapes (Fig. 1A). After osteogenic induction, alizarin red staining showed multiple calcium nodules in the cells, which indicated that the cells had well-developed osteogenic differentiation functions (Fig. 1B). Flow cytometric analysis showed that the HUCMSCs were positive for CD73, CD90 and CD105 but lacked the hematopoietic marker CD45 (Fig. 1C), which was consistent with the characteristics of MSCs.

The characteristics of HUCMSC-exos isolated and purified using a well-established ultracentrifuge method. Our hypothesis was supported by transmission electron microscopy analysis, which showed a cup shape (Fig. 1D). NTA identified particles with diameters of 30–150 nm (Fig. 1E). Furthermore, exosomes were positive for the exosomal protein markers CD63 and CD81 (Fig. 1F). These results indicated that we successfully isolated HUCMSC-exos.

### HUCMSC-exos reduced sFlt-1-induced PE-like adverse birth outcomes in pregnant mice

**Fig. 2:**
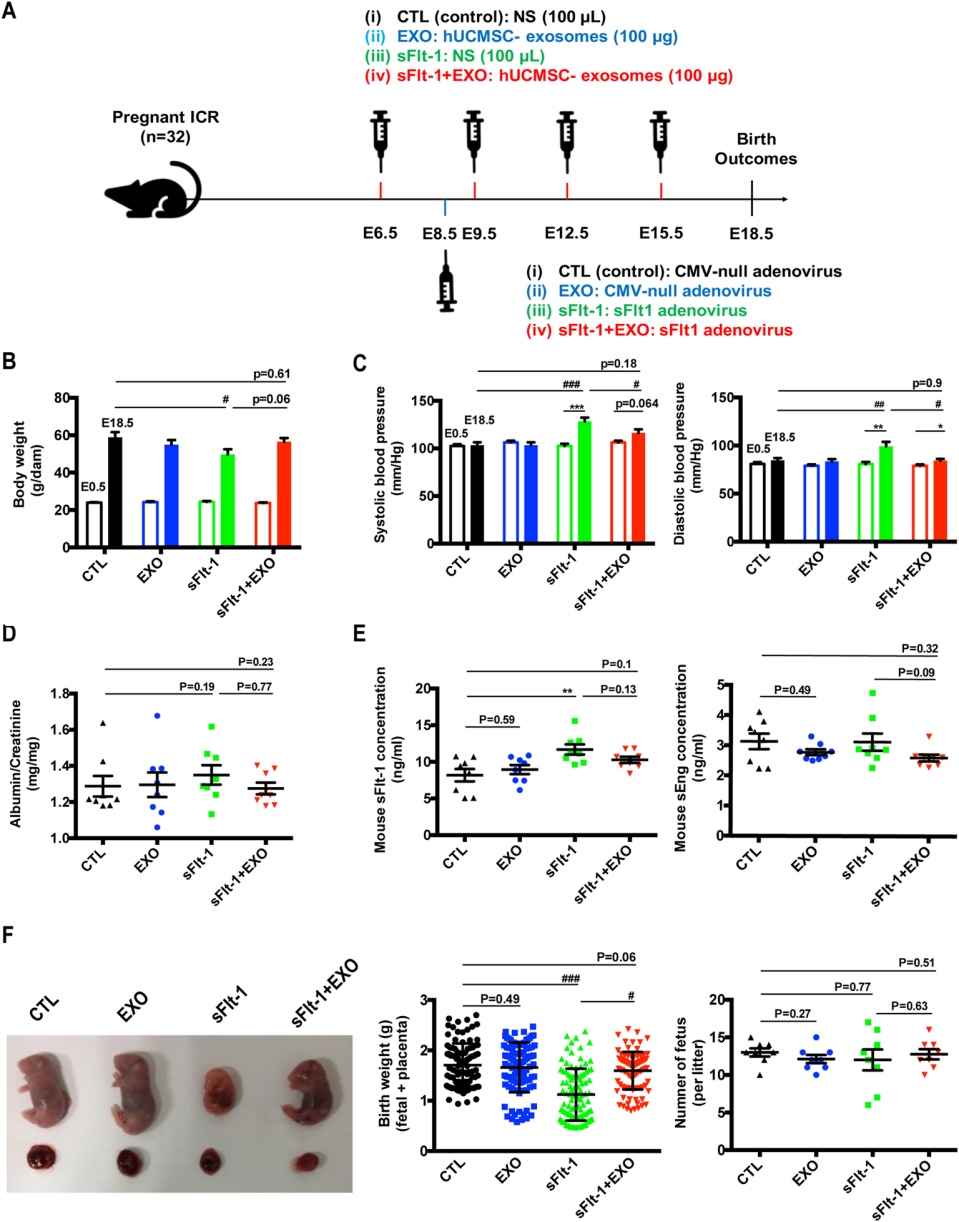
HUCMSC-exos improve the sFlt-1-induced preeclamptic mouse reproductive outcomes. **a** Pregnant mice were randomly divided into four groups and respectively treated with nature saline (i, CTL, *n* = 8, black), HUCMSC-exos (ii, EXO, 100 μg, *n* = 8, green), sFlt-1 adenovirus (iii, sFlt-1, n = 8, blue) or sFlt-1 adenovirus and HUCMSC-exos (iv, sFlt-1 + EXO, 100 μg, *n* = 8, red) injection via tail vein on E6.5, E9.5, E12.5, E15.5 and E15.5. (The syringes above indicated the injection of HUCMSC-exos or nature saline. The syringe below indicated the treatment of sFlt-1 adenovirus or CMV-null adenovirus). To establish PE-like mice model, pregnant mice were injected with CMV-null adenovirus (i, CTL; ii, EXO) and sFlt-1 adenovirus (iii, sFlt-1; iv, sFlt-1 + EXO) on E8.5. **b** Body weights of dams on E0.5 and E18.5 (one-way ANOVA and Dunett's post-hoc tests, sFlt-1 *vs* CTL on E18.5, ^#^p < 0.05). **c** Blood pressure measurements on E0.5 and E18.5 (Paired students’ t-tests, E18.5 *vs* E0.5, *p < 0.05, **p < 0.01, ***p < 0.001; Systolic blood pressure on E18.5, one-way ANOVA and Dunett's post-hoc tests, sFlt-1 *vs* CTL, ^###^*p* < 0.001, sFlt-1 + EXO *vs* sFlt-1, ^#^*p* < 0.05; Diastolic blood pressure on E18.5, sFlt-1 *vs* CTL, ^##^*p* < 0.01, sFlt-1 + EXO *vs* sFlt-1). **d** Ratio of albumin/creatinine in mice urine was measured by ELISA kit on E18.5. **e** Mouse sFlt-1 and sEng concentrations in serum were measured by ELISA kits on E18.5 (one-way ANOVA and Dunett’s post-hoc tests, sFlt-1 *vs* CTL, ^##^*p* < 0.01). **f** Effects of HUCMSC-exos-injection on morphology, birth weights and number of fetuses per litter. (*n* = 8 per group of dams, one-way ANOVA and Dunett's post-hoc tests, sFlt-1 *vs* CTL, ^###^p < 0.001, sFlt-1 + EXO *vs* sFlt-1, ^#^p < 0.05)

We first established the preeclampsia-like mice model by injecting the sFlt-1 adenovirus into pregnant ICR mice via the tail vein on E8.5. HUCMSC-exos were administered to examine the potential of exosomes in treating PE. Pregnant mice were injected with exosomes or diluent on E6.5, E9.5, E12.5 and E15.5 (Fig. 2A). Hypertension is the most common diagnostic sign of PE, then blood pressure response in the sFlt-1-induced preeclampsia model was examined and shown in Fig. 2C. The sFlt-1-injected mice had both increased systolic and diastolic blood pressure. No significant differences were observed among the CTL and EXO groups, while dams treated with both sFlt-1 and HUCMSC-exos remained nearly normotensive on E18.5 relative to that of dams treated only with sFlt-1. SFlt-1 injection induced the production of sFlt-1 and increased its serum concentration in mice (Fig. 2E, sFlt-1 vs CTL, *P* < 0.01) without affecting sEndoglin (sEng) levels. Circulating sFlt-1 concentrations were slightly decreased by exosome treatment (sFlt-1 vs EXO + sFlt-1, *P* = 0.13). Interestingly, dams treated with both sFlt-1 and HUCMSC-exos showed slightly reduced sEng level (sFlt-1 vs EXO + sFlt-1, *P* = 0.09), thus further studies need to be explored.

The effects of exosomes on PE-induced IUGR were evaluated, and the observed decrease in body weight (Fig. 2B) was most likely caused by small-sized fetuses and placentas rather than a reduction in the number of fetuses (Fig. 2F). Fetuses that survived to term had decreased weights and were small in the sFlt-1-treated group, and exosomes prevented this growth restriction in fetuses and placentas (Fig. 2F). The urine albumin/creatinine ratio (ACR) was slightly increased after sFlt-1 injection, but the difference was not significant (Fig. 2D *P*=0.19). These results suggest that HUCMSC-exos significantly reduce the IUGR that occurs in the fetuses of dams exposed to sFlt-1. Therefore, HUCMSC-exos have the potential to treat PE by improving sFlt-1-induced hypertension and IUGR in mice.

### HUCMSC-exos improved placental vascular development in PE-like mice

**Fig. 3:**
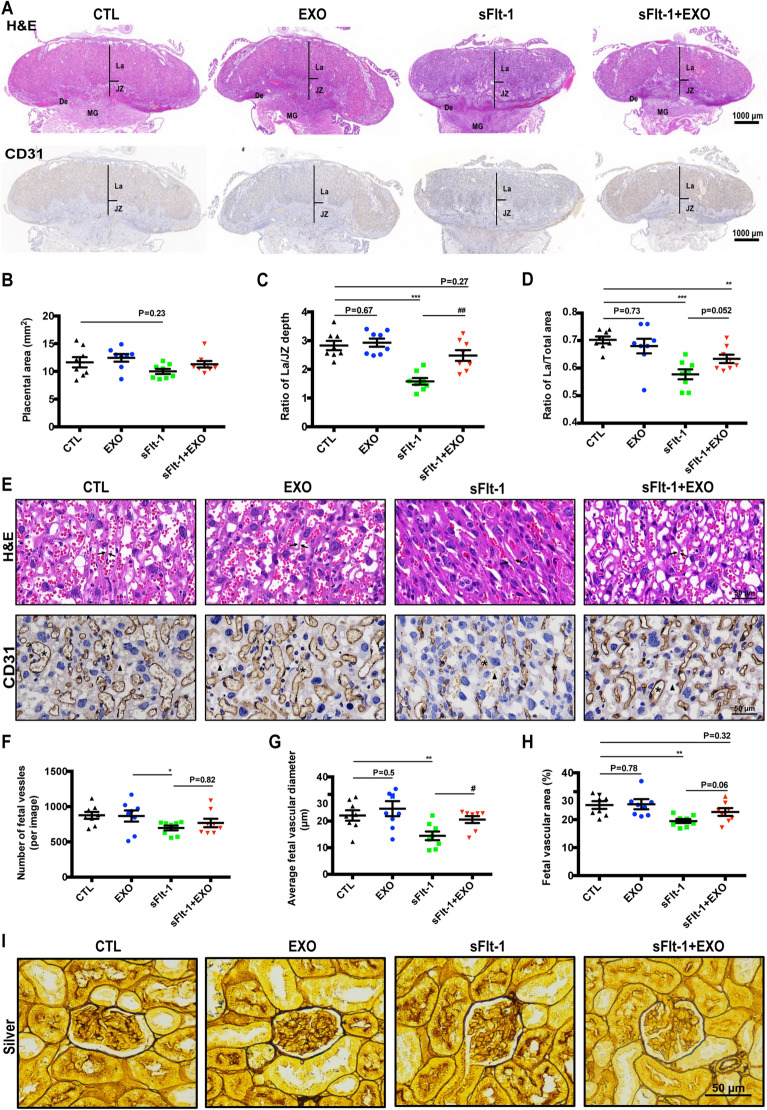
Effects of HUCMSC-exos on histopathology of mice placenta and kidney. **a** H&E staining and IHC staining for CD31 of mice placentas (JZ, jountional zone; L, labyrinth), scale bar 2000 μm. **b** Placental area of pregnant mice. **c** Ratio of La/JZ depth of placenta. **d** Ratio of La/total area of placenta. **e** H&E and IHC staining for CD31 in mice placental tissues. Brown color indicates positive staining for CD31. Arrowheads: trophoblastic septa; asterisk: fetal vascular; triangle: intervillous space. Scale bar 50 μm. Numbers of fetal vessles (**f**), average fetal vascular diameter (**g**) and fetal vascular area (**h**) in JZ were measured (*n* = 8 per group of dams, one-way ANOVA and Dunett’s post-hoc tests, * sFlt-1 *vs* CTL, *p < 0.05, **p < 0.01, ^#^ sFlt-1-exo *vs* sFlt-1, ^#^*p* < 0.05). **i** Staining of kidney sections with PAS, scale bar 50 μm

The sFlt-1 virus-treated mice showed retarded fetal development, which is driven by impaired placental feto-maternal exchange. Thus, we examined the histology of the placentas, including the labyrinth (La) and junctional zone (JZ) by H&E and IHC staining (Fig. 3A). The total placental area did not change among the four groups (Fig. 3B), while the thickness of the La layer was notably reduced in sFlt-1-injected mice and rescued by HUCMSC-exo (Fig. 3C, D). Then, we analyzed placental vascularization by staining for CD31, which specifically binds to endothelial cells. High magnification views showed extensive vascular damage in the La, which indicated impaired blood flow, in the sFlt-1 group. Importantly, in EXO + sFlt-1 mice, there was a partial reversal of the vascular narrowing observed in placentas from sFlt-1 mice, as indicated by increased lumen diameters and areas (Fig. 3E). The number of lumens per image in the exosome-treated group relative to the sFlt-1 group was slightly increased but was not significant (Fig. 3F). Quantification of vascular diameter after CD31 staining showed that this decreased from 22.1 ± 1.95 μm in CTL mice to 14.45 ± 1.64 μm in sFlt-1 mice (*P* < 0.01) and reversed to 21.6 ± 1.44 μm in EXO + sFlt-1 mice (P < 0.01) (Fig. 3G). Furthermore, the fetal vascular area (%) was reduced in sFlt-1 mice (19.49 ± 0.71 vs 25.23 ± 1.38, *p* < 0.001) and increased to 22.75 ± 1.4 in EXO + sFlt-1-treated mice (*p* = 0.06) (Fig. 3H). There was no significant difference in the vascular number, diameter, or area between EXO and CTL mice. Neither glomerular endotheliosis nor mesangial expansion, which are characteristic features of PE, was present in any group, as determined by PAS stain (Fig. 3I).

### In vitro proangiogenic effects of HUCMSC-exos

**Fig. 4:**
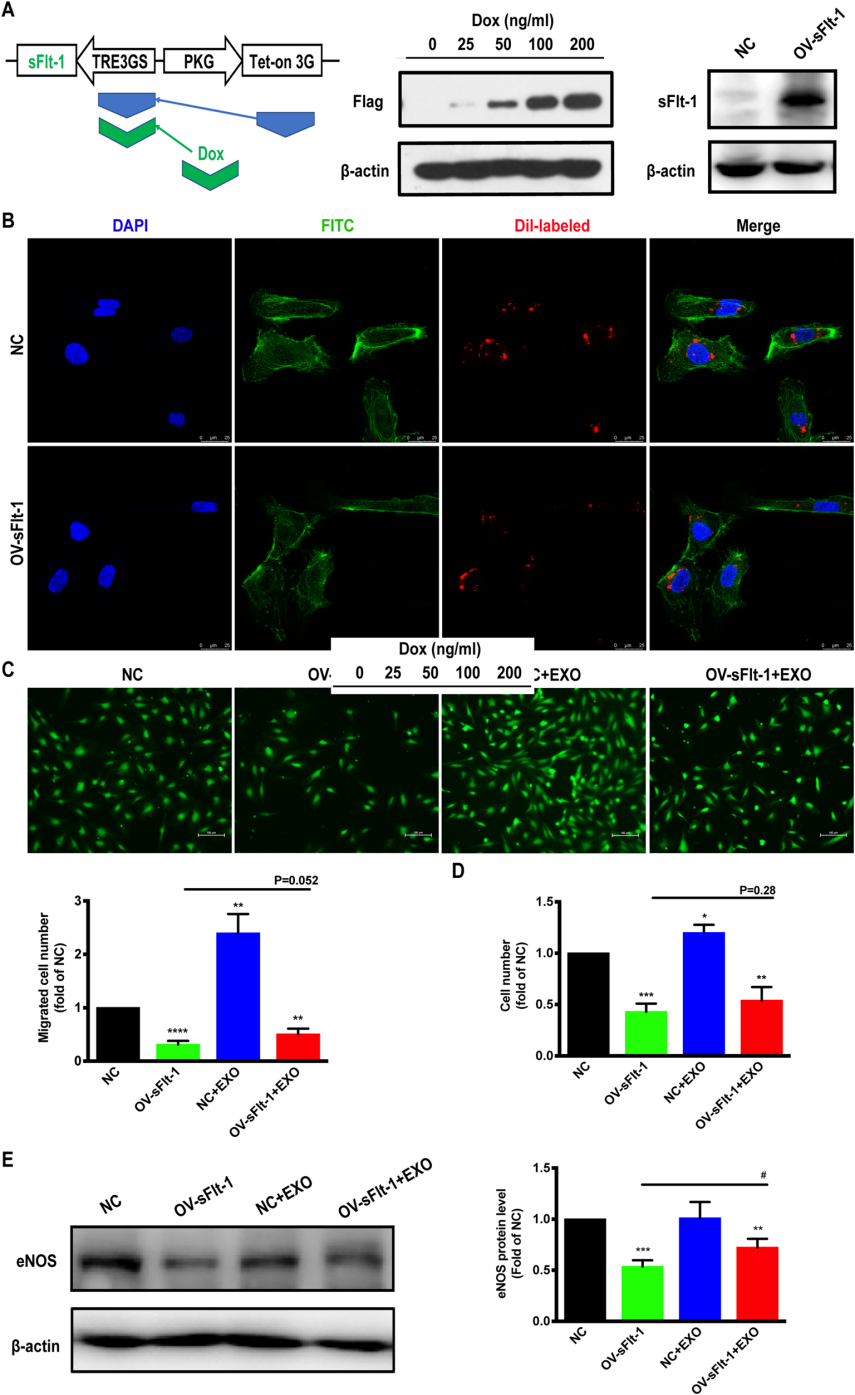
Proangiogenic effects of HUCMSC-exos. **a** Stably transfected HUVECs were treated with the indicated concentrations of Dox for 24 h. Western blot analysis of sFlt-1 in HUVECs was performed using anti-Flag and anti-sFlt-1 antibody. **b** NC-HUVECs and OV-sFlt-1-HUVECs were incubated with dil-labeled HUCMSC-exos (red) for 24 h before fluorescent and phase contrast images were captured, scale bar 20 μm. **c** Cell migration response to HUCMSC-exos (100 μg/mL) was determined by transwell assay, scale bar 100 μm. **d** After incubation with HUCMSC-exos (100 μg/mL), HUVEC proliferation was analyzed by CCK-8 assay. **e** Protein expression of eNOS in HUVECs were detected by western blot. All data were means ± S.E.M. of 3 pairs of independent experiments performed. One-way ANOVA, *p < 0.05, **p < 0.01, ***p < 0.001

A substantial body of evidence indicates that PE in humans is a consequence of vascular endothelial damage, resulting in multiorgan dysfunction. Therefore, we chose HUVECs as the cell model for studying the effects of HUCMSC-exos on endothelial dysfunction in PE. The Tet-On-sFlt-1_Flag_ HUVEC model was established to simulate vascular endothelial cells in women with PE. In the absence of Dox, negative control (NC) HUVECs did not express any detectable sFlt-1 protein, and following induction with different doses of Dox, strong signals for sFlt-1 were observed in sFlt-1-overexpressing (OV-sFlt-1) cells, as measured by western blotting (Fig. 4A). HUCMSC-exos labeled with the fluorescent dye Dil were incubated with NC and OV-sFlt-1 HUVECs for 24 h, and most recipient cells were positive for Dil fluorescence and showed no difference among the groups (Fig. 4B).

A series of cellular functional analyses were performed, and we first demonstrated that HUCMSC-exos are capable of facilitating angiogenesis because they contain many proangiogenic components. OV-sFlt-1-HUVEC proliferation and migration were significantly inhibited, and these attenuated angiogenetic behaviors were reversed after incubation with HUCMSC-exos (Fig. 4C, D). It is suggested that high levels of circulating sFlt-1 lead to suppression of eNOS signaling pathway, which, in turn, enhances sensitivity to vasoconstrictors that induces maternal hypertension in PE. Therefore, we examined the protein expression of eNOS in HUVECs and as expected, found that OV-sFlt-1-HUVECs exhibited decreased eNOS protein expression, which was partially rescued in the presence of HUCMSC-exos (Fig. 4E). These findings suggest that HUCMSC-exos have the potential protective effects on sFlt-1-induced endothelial and eNOS dysfunction.

### Proteomic analysis of HUCMSCs and HUCMSC-exos

**Fig. 5:**
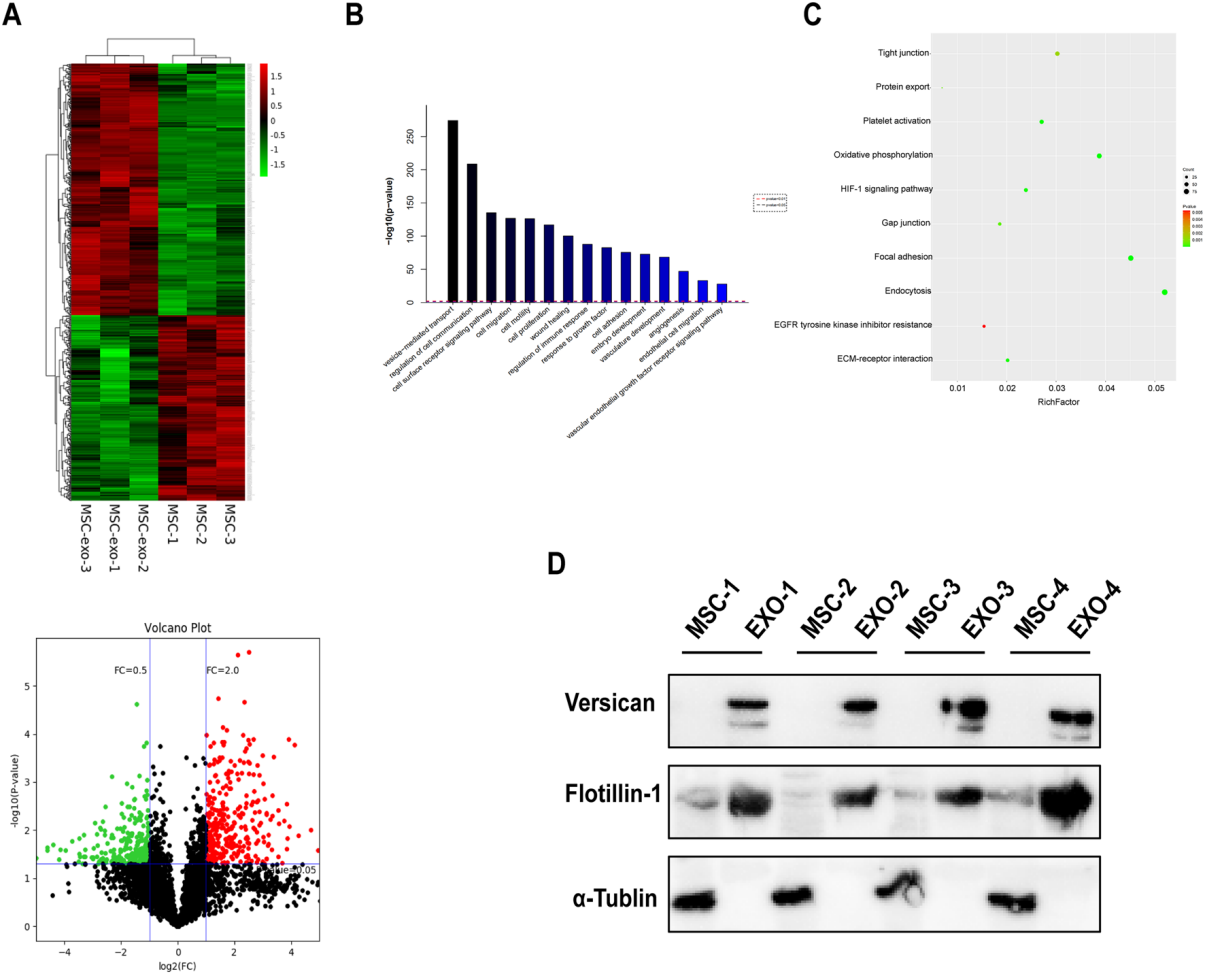
Proteomic analysis of HUMSC and HUCMSC-exos. **a** Cluster analysis of differentially expressed genes. The colors from green to black and to red represent the expression values of the differentially expressed genes that have increased. Volcano plots for HUCMSCs *vs* HUCMSC-exos. Statistically significant proteins (*P* < 0.05) with a log2 fold change + 2 or − 0.5 are represented by red or green dots, respectively. Differentially expressed proteins that did not reach statistical significance are represented by black dots. Gene Ontology (GO) analysis of the top pathways (**b**) and bubble diagram of KEGG pathway enrichment (**c**) in both HUCMSC and HUCMSC-exos. (**d)** Protein expressions of Versican, Flotillin-1, and α-Tublin in HUCMSCs and HUCMSC-exos were detected by western blot

HUCMSC-exos modulated sFlt-1-induced endothelial dysfunction and improved placental angiogenesis in PE-like mice. It was, therefore, decided to investigate how exosomes enhanced vascular angiogenesis. After successfully extracting proteins from exosomes and parallel cells, proteomics analysis using tandem mass tag (TMT) technology was performed to detect protein expression profiles. Unsupervised hierarchical clustering analysis was used to generate a heat map of the differentially expressed proteins, and a total of 568 proteins were differentially expressed between HUCMSC-exos and HUCMSCs using the cutoff value of a two-fold change and a P-value < 0.05 (Fig. 5A). On the basis of gene ontology (GO) enrichment analysis, both HUCMSCs and HUCMSC-exos displayed functional enrichment in biological processes related to vesicle-mediated transport, cell communication, cell migration, and angiogenesis (Fig. 5B). Kyoto Encyclopedia of Genes and Genomes (KEGG) pathway enrichment analysis is shown in Fig. 5C. The degree of gene enrichment was represented by abscissa, the amount of gene enrichment was represented by the bubble size, and p-value was represented by the color depth. The main pathways of enrichment included tight junction, protein export, platelet activation, oxidative phosphorylation, HIF-1 signaling pathway, gap junction, focal adhesion, endocytosis, EGFR tyrosine kinase inhibitor resistance and extracellular matrix–receptor (ECM-receptor). We next analyzed the differentially expressed genes (DEGs) between these two chambers. Compared with HUCMSCs, the up-expressed proteins in HUCMSC-exos were MMP2 (Matrix metalloproteinase 2, fold change = 14.7) and VCAN (versican, fold change = 11.7). Previous study showed that MSC-exo could promote the migration of endothelium cells by delivering MMP2. Then, we verified that VCAN, which contributes to tissue development and maintenance by participating in cell adhesion, proliferation and angiogenesis via binding to ECM components, was obviously upregulated in HUCMSC-exos compared with cell lysates (Fig. 5D).

## Discussion

PE is a unique multisystem disorder that leads to maternal and fetal morbidity and mortality. Therapeutic strategies aimed at reducing blood pressure and improving fetal birth outcomes are being pursued as avenues to prevent PE [24]. In the current study, we showed that HUCMSC-exos provided therapeutic benefits in animal models of PE by improving birth outcomes and vascular functions.

Compelling evidence indicates that MSCs and their derived exosomes can be used in many vascular dysfunction-related diseases [25]. Due in part to studying the paracrine effects of stem cell therapy, this specific therapy has been mechanistically linked to the inherited functions of secreting exosomes [26]. Recently, research suggested HUCMSC-originated exosomes might be an innovative direction for therapeutic approaches against PE by accelerating trophoblast cell invasion [27]. In addition, HUCMSC-exos-mediated angiogenesis by promoting the expression of vascular endothelial growth factor (VEGF) and Angiopoietin 1 has been reported [28]. Mechanistic studies revealed MSC-derived exosomal miR-21 targets the NOTCH-1/Delta-like-4 pathway to enhance angiogenesis [29]. In the current study, we chose a well-established mouse model of PE that mimics many features of the human pregnancy disease, including upregulated blood pressure and serum sFlt-1 concentration. In accordance with our previous findings, exosomes from human fluids can enter murine organs including placenta, thus we focused on HUCMSC-exos as potential cell-free therapeutics for the treatment of PE. Administration of HUCMSC-exos prevented the development of sFlt-1-induced preeclamptic complications by decreasing blood pressure and improving fetal and placental weights. Circulating sFlt-1 levels are considered a diagnostic and prognostic marker of PE, and sFlt-1 is a clinically promising therapeutic target for this disease [30]. In our mouse model, administration of HUCMSC-exos slightly decreased the serum sFlt-1 concentration.

After exchange of gases and nutrients between the maternal and fetal circulation in the maze of labyrinth (La), deoxygenated blood then moves to the junctional zone (JZ) [31]. Widespread vascular damage, insufficient placental vascularization and reduced blood flow to the feto-placental unit resulted in impaired fetal growth in PE [32]. We demonstrated that placental vascular network density in the La zone of sFlt-1-treated mice was sparse, and the fetal sinusoids were narrow. The mice treated with HUCMSC-exos had improved fetal and placental weight and size as the result of a decrease in the diffusion distance between the fetal and maternal blood supplies that provided enough nutrition for the developing fetus. Despite the therapeutic effects observed, however, the underlying molecular mechanism, especially how the vascular is regulated, remains unclear. Then, we indicated the addition of HUCMSC-exos rescued cell proliferation and migration abilities of OV-sFlt-1-HUVECs in vitro. NO-mediated reductions in blood pressure are crucial, and decreased NO bioavailability can lead to hypertension by enhancing vascular oxidative stress and endothelial dysfunction [33]. OV-sFlt-1-mediated interference in eNOS expression in HUVECs could be reversed by exosomes. Therefore, HUCMSC-exos may serve as a novel medication that rescues endothelial dysfunction in treating PE.

Exosomes have therapeutic potential owing to their roles as carriers to deliver nucleic acids and proteins between human body systems [34]. Genetic or molecular engineering of exosomes can improve target specificity and anti-disease activity with less toxicity [35]. Based on our proteomic analysis, a better understanding of the molecular properties of HUCMSC-exos contributes to their therapeutic potential as innovative drug delivery systems. Previous study showed that MSC-exo could promote the migration of endothelium cells by delivering MMP2 [36]. Exosomal MMP2 was demonstrated to promote endothelial angiogenesis via VEGF/Erk1/2 signaling pathway [37]. VCAN, which highly expressed in metabolically active tissues [38] can mediate angiogenesis possibly depends upon interactions with VEGF and influence the assembly of the ECM [39]. Upon uptake by the vascular endothelium, these proteins accumulated in HUCMSC-exos promoted cell proliferation, migration and angiogenesis to rescue damaged vascular tissues in preeclamptic-like mice.

Here, we provide new evidence that HUCMSC-exos have the ability to reverse sFlt-1-induced PE-like adverse birth outcomes in vivo. In addition, exosomes can facilitate sFlt-1-imparied endothelial dysfunction in HUVECs. The proteomic analysis revealed that HUCMSC-exos were highly enriched in the proteins involved in regulation of cell communication, cell migration, and angiogenesis, as well as vascular endothelial growth factor receptor signaling pathway. Based on the improvements brought about by HUCMSC-exos treatment in terms of vascular functions and birth quality in PE-like mice model, we propose that HUCMSC-exos may represent a new approach to treat or prevent PE.

## Conclusion

In summary, we revealed the VCAN were highly expressed in exosomes derived from HUCMSCs. Additionally, we reported the beneficial effects of HUCMSC-exos in sFlt-1-induced preeclamptic mice by promoting angiogenesis. Thus, we provide new evidence for the consideration of HUCMSC-exos as a novel therapeutic approach to PE treatment.

## Data Availability

The raw data are available from the authors on reasonable request.

## Abbreviations

HUCMSCs: Human umbilical cord mesenchymal stem cells
sFlt-1: Soluble fms-like tyrosine kinase-1
HUCMSC-exos: Human umbilical cord mesenchymal stem cell-derived exosomes
HUVEC: Human umbilical vein endothelial cell
PE: Preeclampsia
IUGR: Intrauterine growth retardation
MHC-II: Major histocompatibility complex-II
FBS: Fetal bovine serum
ECGS: Endothelial cell growth supplement
BSA: Bovine serum albumin
DOX: Doxycycline
TEM: Transmission electron microscope
NTA: Nanoparticle tracking analysis
H&E: Hematoxylin and eosin
PAS: Periodic acid-methenamine silver
NC: Negative control
TMT: Tandem mass tag
ANOVA: Analysis of variance
ACR: Albumin/creatinine ratio
La: Labyrinth
JZ: Junctional zone
MMP2: Matrix metalloproteinase 2
ECM: Extracellular matrix
VCAN: Versican

## Data availability

All relevant data are within the paper.
